# Perfluoroalkyl substances exposure in early pregnancy and preterm birth in singleton pregnancies: a prospective cohort study

**DOI:** 10.1186/s12940-020-00616-8

**Published:** 2020-06-03

**Authors:** Xiaona Huo, Lin Zhang, Rong Huang, Liping Feng, Weiye Wang, Jun Zhang, for the Shanghai Birth Cohort

**Affiliations:** 1 MOE-Shanghai Key Laboratory of Children’s Environmental Health, Xinhua Hospital, Shanghai Jiao Tong University School of Medicine, 1665 Kong Jiang Road, Shanghai, 200092 China; 2 Obstetrics Department, International peace maternity and child health hospital of China, Shanghai Jiao Tong University School of Medicine, 910 Hengshan Road, Shanghai, 200030 China; 3 School of Public Health, Shanghai Jiao Tong University School of Medicine, Shanghai, 200052 China

**Keywords:** Birth cohort, Perfluoroalkyl substances, Pregnancy, Duration of gestation, Preterm birth

## Abstract

**Background:**

Preterm birth (PTB, < 37 completed weeks’ gestation) is one of the global public health concerns. Epidemiologic evidence on the potential impact of perfluoroalkyl substances (PFAS) on PTB is still limited and inconsistent. We aimed to investigate the associations between prenatal PFAS exposure and PTB among singleton live births.

**Methods:**

We studied 2849 mother-infant pairs in the Shanghai Birth Cohort (SBC) from 2013 to 2016. Ten PFAS in maternal plasma in early pregnancy (gestational age, median (interquartile range): 15 (13–16) weeks) were measured. Primary outcomes were duration of gestation, PTB, spontaneous PTB and clinically indicated PTB. A linear regression model was used to assess the associations between ln-transformed PFAS and duration of gestation (in weeks). Logistic regression models were applied to estimate the relative risks of these outcomes.

**Results:**

The incidence of overall PTB was 4.8% (95% confidence limit: 4.0–5.6%, *n* = 136) in this study population. In the linear regression analyses, PFAS were not associated with the duration of gestation after controlling for potential confounders. In the multiple logistic models, no significant associations were observed between PFAS and overall PTB, spontaneous or indicated PTB.

**Conclusion:**

In this prospective cohort study, we did not observe significant associations between maternal plasma PFAS concentrations in early pregnancy and gestational length, overall PTB, spontaneous or indicated PTB.

## Background

Preterm birth (PTB), defined as a birth < 37 completed weeks’ gestation, is one of the global public health problems with an average rate of 10.6% around the world [1, 2]. PTB is the leading cause of neonatal mortality and under-5 mortality worldwide in 2016 [1]. Children with PTB are at increased risks of long-term consequences such as impaired neurodevelopment, respiratory complications and gastrointestinal diseases [3]. Despite great efforts to reduce PTB [4, 5] in many countries, the incidence has not yet been significantly reduced. PTB is a particular concern in China as China has a minimum of 1–1.5 million preterm births each year and the incidence has been increasing in the past 2–3 decades [1, 5–7].

Environmental pollution, endocrine disrupting chemicals in particular, has been suspected to be one of the culprits [8]. For example, perfluoroalkyl substances (PFAS) are a subgroup of the widespread synthetic chemicals and persistent in the environment [9]. PFAS have excellent oil-repellent, water-repellent and dirt resistance characteristics [10]. They have been extensively used in household products (e.g., non-stick cookware, shampoo and carpets) since the 1950s [11]. Humans are exposed through oral ingestion, skin contact and inhalation [12].

Previous studies demonstrated that PFAS could change lipid metabolism [13], disrupt thyroid function [14], interfere glucose tolerance [13], and affect estrogen homeostasis [15]. It is, therefore, plausible that PFAS may cause PTB. However, epidemiologic evidence linking PFAS with PTB or length of gestation is limited and inconsistent. Two birth cohort studies in Denmark and U.S. reported that the estimated risk for PTB in the higher quartiles of maternal perfluorooctanote (PFOA) and perflourooctanesulfonate (PFOS) exposure in early pregnancy was increased by 1.7 fold or more comparing to the lowest quartile [16, 17]. In addition, nearly 1.5-fold odds of PTB was found for per doubling increase of perfluoroheptane sulfonate (PFHpS) and perfluorodecanoic acid (PFDA) concentrations in the Danish study [17]. A 1.2-fold risk of PTB was reported for per interquartile range of perfluorononanoic acid (PFNA) in the U.S. study [16]. A study in Taiwan, China, also found that a higher level of PFOS but not PFOA in the cord blood was associated with an increased risk of PTB (odds ratio: 2.5, 95% CI: 1.5, 4.1) [18]. In contrast, several other studies did not observe any significant associations [19–22].

The current study aimed to examine the associations between PFAS in early pregnancy and duration of gestation and risk of PTB in a large prospective cohort in Shanghai, China.

## Methods

### Study population

The study subjects in the current analysis were from the Shanghai Birth Cohort, a large prospective study in Shanghai, China, from 2013 to 2016 [23]. The inclusion criteria were couples aged 20 years or older, married, seeking preconception care or in early pregnancy, with spontaneous conception, at least one of them being registered residents in Shanghai with no plan to move out of Shanghai for 2 years after enrollment. The participants provided an informed consent before enrollment, and were interviewed by trained staffs to complete structured questionnaires on demographic characteristics, previous reproductive and medical history and lifestyle factors. They also provided blood samples voluntarily at the recruitment visit. Clinically relevant characteristics of the current pregnancy such as pregnancy complications, chronic diseases during current pregnancy, number of fetus, infant sex and gestational age at birth were extracted from medical records. An ethical approval was obtained from the Ethical Committee of the Xinhua Hospital affiliated to the Shanghai Jiao Tong University School of Medicine.

For the current analysis, a total of 3242 women who provided blood samples in early pregnancy (gestational age, median (interquartile range; IQR): 15 (13–16) weeks) and delivered a singleton live birth were eligible. Births with missing information on gestational age at birth, gestational age < 20 weeks or > 42 weeks were excluded (*n* = 289). Mothers or infants with missing information on maternal age at enrollment, pre-pregnancy body mass index (BMI), parental educational level, parity, chronic diseases during pregnancy or infant sex were excluded (*n* = 104). Finally, 2849 mother-infant pairs were included for analysis.

### PFAS measurement

Blood samples were taken during early pregnancy and immediately centrifuged and frozen at − 80 °C. A detailed analytical method has been described elsewhere [24]. In brief, PFAS concentrations were measured in 100 μl plasma using high-performance liquid chromate graphy/tandem mass spectrometry (HPLC/MS-MS; Agilent1290–6490, Agilent Technologies Inc., USA). The intra- and inter-assay coefficients of variation were both below 10%. The limit of detection (LOD) was 0.09 ng/mL for PFOS and PFOA, 0.05 ng/mL for perfluorododecanoic acid (PFDoA), 0.03 ng/mL for perfluoroheptanoic acid (PFHpA), 0.02 ng/mL for PFDA, PFNA, perfluoroundecanoic acid (PFUA) and perfluorohexanesulfonate (PFHxS), 0.12 ng/mL for perfluorooctane sulfonamide (PFOSA), and 0.009 ng/mL for PFBS.

### Outcome

The main outcome of this study was PTB, which was defined as birth between 21 and 36 completed weeks’ gestation. Spontaneous PTB referred to spontaneous onset labor and preterm premature rupture of the membranes (PPROM) irrespective of mode of delivery (vaginal or cesarean section), while clinically indicated PTB was defined as PTB for preeclampsia, fetal stress, placenta previa and other maternal, fetal or placenta indications [3].

### Covariates

Demographic characteristics included maternal age (< 30, 30–34, ≥ 35 years old), parental education levels (≤ 12, > 12 years), smoking during pregnancy (no, yes, unknown), gestational age at blood collection. Obstetric and medical covariates included pre-pregnancy BMI (< 18.5, 18.5–24.9, ≥ 25 kg/m^2^), parity (0, ≥ 1), pregnancy complicated by chronic diseases (no, yes), and infant sex (female, male). Chronic diseases included heart, kidney or liver disease, diabetes mellitus, chronic hypertension disorders, epilepsy, malnutrition and anemia.

### Statistical analysis

Distributions of demographic and pregnancy related characteristics were showed as numbers and percentages. PFAS with concentrations below the LOD were assigned LOD/ √2. The distribution of PFAS in early pregnancy was presented as medians and interquartile ranges. Multivariable restricted cubic spline model was used to examine potential non-linear relationships between PFAS and gestational age (in weeks) at birth, according to the ln-transformed PFAS at three knots of 10th, 50th, 90th percentile. Univariable and multivariable linear regression analyses were used to evaluate the associations of ln-transformed PFAS with gestational age (in weeks) at birth. Simple and multiple logistic regression analyses were applied to estimate odds ratios (ORs) and 95% confidence intervals (CIs) of the overall PTB, spontaneous PTB and clinically indicated PTB. In the logistic regression models, PFAS were entered as continuous variables (ln-transformed) and in tertile (untransformed) with the lowest tertile served as the reference group. Based on the directed acyclic graphs and existing literature, covariates were included in all adjusted models, including maternal age (years), pre-pregnancy BMI (kg/m^2^), parental education levels (≤ 12, > 12 years), parity (0, ≥ 1), chronic diseases (no, yes), infant sex (female, male) and gestational week at blood collection. Because only few mothers (*n* = 9, 0.3%) smoked during early pregnancy, maternal smoking status was not included in the adjusted models.

To investigate the potential modification of infant sex, we performed a stratified analysis. To examine whether the associations differed among women who were multiparous, had preterm labor history, or complicated by chronic diseases, we carried out several subgroup analyses, restricting to women without these conditions, respectively.

Spearman’s rank correlation coefficient was used to estimate the correlations between each pair of PFAS. As multicollinearity between PFAS may bias the estimated effects, we conducted linear regression or logistic regression models with mutual adjustment of the correlated PFAS (*ρ* > 0.4).

All analyses were carried out using SAS version 9.4 (IBM SAS Institute Inc., Cary, NC) and R software (R version 3.6.1). Results at *p* < 0.05 were thought to be statistically significant.

## Results

Of all the 2849 women included in the present study, 136 (4.8%) gave a singleton live birth before 37 weeks’ gestation, including 97 (71.3%) spontaneous PTB and 39 (28.7%) indicated PTB. More than 60% of the women aged less than 30 years and most of them were nulliparous (85.5%), nonsmokers (99.4%) and had normal weight (18.5 < BMI < 25 kg/m^2^) before pregnancy (74.4%). The vast majority (95.0%) of couples had more than 12 years of education and only 196 (6.9%) women complicated with chronic diseases during pregnancy. In addition, the rate of boys and girls was 51.1 and 48.9%, respectively (Table 1).

**Table 1:** Demographic and reproductive characteristics, *n* = 2849

Characteristics	n (%)
**Maternal age (years)**	
< 30	1811 (63.6)
30–34	845 (29.7)
≥ 35	193 (6.8)
**Pre-pregnancy BMI**^a^**(kg/m**^**2**^**)**	
< 18.5	435 (15.3)
18.5–24.9	2119 (74.4)
≥ 25	295 (10.4)
**Parental educational level (years)**	
≤ 12	142 (5.0)
> 12	2707 (95.0)
**Smoking status in early pregnancy**	
No	2831 (99.4)
Yes	9 (0.3)
Unknown	9 (0.3)
**Parity**	
0	2435 (85.5)
≥ 1	414 (14.5)
**Pregnancy complicated by chronic diseases**^a^	
No	2653 (93.1)
Yes	196 (6.9)
**Infant sex**	
Male	1456 (51.1)
Female	1393 (48.9)
**Gestational age at blood drawn (weeks)**	
Median (IQR)	15 (13–16)

^a^*BMI* body mass index; Chronic diseases including heart disease, kidney or liver disease, diabetes mellitus, chronic hypertension disorders, epilepsy, malnutrition and anemia; *IQR* interquartile range

PFOA, PFOS, PFNA, PFDA, PFUA and PFHxS were detected in all samples, whereas PFHpA, PFBS, PFDoA and PFOSA were quantified in 82.6, 81.2, 83.1 and 19.6% of samples, respectively. PFOSA was not included in following analyses for a low detection rate (< 80%). Median concentrations were 11.85, 9.33, 1.69, 1.69. 1.39, 0.54, 0.16, 0.06 and 0.04 ng/mL for PFOA, PFOS, PFNA, PFDA, PFUA, PFHxS, PFDoA, PFHpA and PFBS, respectively (Table 2). Most PFAS generally showed various degrees of correlation between each other (Table S1).

**Table 2:** The distribution of plasma concentrations of PFAS (ng/mL) at the enrollment (*n* = 2849)

PFAS	LOD	% < LOD		25th percentile	Median	75th percentile
PFOA	0.09	0		9.20	11.85	15.26
PFOS	0.09	0		6.54	9.33	13.65
PFDA	0.02	0		1.14	1.69	2.52
PFUA	0.02	0		0.94	1.39	2.05
PFNA	0.02	0		1.21	1.69	2.36
PFHxS	0.02	0		0.42	0.54	0.69
PFHpA	0.03	17.4		0.04	0.06	0.10
PFBS	0.009	18.8		0.02	0.04	0.06
PFDoA	0.05	16.9		0.10	0.16	0.25
PFOSA	0.12	80.4		–	–	–

Note: *LOD* limit of dection, *PFOA* perfluorooctanate, *PFOS* perfluorooctane sulfonate, *PFDA* perfluorodecanoic acid, *PFUA* perfluoroundecanoic acid, *PFNA* perfluorononanoic acid, *PFHxS* perfluorohexanesulfonate, *PFHpA* perfluoroheptanoic acid, *PFBS* perfluorobutane sulfonate, *PFDoA* perfluorododecanoic acid, *PFOSA* perfluorooctane sulfonamide

Non-linearity was not detected for the association between any ln-transformed PFAS and gestational age at birth (in weeks) in a spline model (see Figure S1). No significant associations between ln-transformed PFAS and gestational age at birth (in weeks) were observed in linear regression after adjusting for potential confounders (Table 3). In the multiple logistic regression analyses, the estimated associations between PFAS and overall PTB, spontaneous PTB, and indicated PTB were non-significant when PFAS treated as continuous ln-transformed variables (Table 4). After categorizing PFAS into tertile variables, PFOS significantly decreased the risks of overall PTB (adjusted OR (aOR): 0.61, 95% CI: 0.40, 0.94) and spontaneous PTB (aOR: 0.56, 95% CI: 0.34, 0.94) comparing the middle tertile with the lowest tertile (see Table S2). However, the statistical significance disappeared after adjusting for correlated PFAS (data were not shown).

**Table 3:** Associations between PFAS concentrations in early pregnancy and gestational age (in weeks) at birth

PFAS (ng/ml)	β (95% CI)	Adjusted^a^ β (95% CI)
PFOA	0.00 (−0.13, 0.14)	−0.00 (−0.14, 0.13)
PFNA	0.03 (−0.07, 0.14)	0.03 (−0.08, 0.13)
PFDA	0.03 (− 0.06, 0.12)	0.04 (− 0.05, 0.13)
PFUA	0.07 (− 0.02, 0.16)	0.06 (− 0.03, 0.15)
PFOS	−0.02 (− 0.12, 0.08)	0.02 (− 0.08, 0.12)
PFHxS	− 0.02 (− 0.15, 0.10)	−0.02 (− 0.14, 0.11)
PFHpA	0.02 (− 0.04, 0.07)	0.02 (− 0.05, 0.08)
PFBS	− 0.01 (− 0.09, 0.07)	0.02 (− 0.05, 0.10)
PFDoA	0.10 (− 0.01, 0.20)	0.08 (− 0.02, 0.19)

*Note*: PFAS concentrations were measured in maternal plasma and have been ln-transformed before entering into the model

^a^Adjusted for maternal age (years), pre-pregnancy BMI (kg/m^2^), parity (0, ≥ 1), parental educational levels (≤ 12, > 12 years), pregnancy complicating with chronic diseases (no, yes), infant sex (male, female) and gestational age at blood drawn (weeks)

**Table 4:** Associations between PFAS concentrations in early pregnancy and preterm birth

PFAS (ng/ml)	Overall preterm birth		Spontaneous preterm birth		Indicated preterm birth	
	N_preterm_ = 136/N_term_2713		N_preterm_ = 97/N_term_ = 2713		N_preterm_ = 39/N_term_ = 2713	
	OR (95% CI)	aOR^a^ (95% CI)	OR (95% CI)	aOR^a^ (95% CI)	OR (95% CI)	aOR^a^ (95% CI)
PFOA	0.93 (0.62, 1.40)	0.92 (0.61, 1.33)	0.74 (0.45, 1.20)	0.73 (0.45, 1.19)	1.63 (0.78, 3.39)	1.71 (0.80, 3.67)
PFNA	0.85 (0.61, 1.18)	0.86 (0.61, 1.20)	0.81 (0.55, 1.19)	0.82 (0.56, 1.22)	0.94 (0.52, 1.71)	0.99 (0.53, 1.86)
PFDA	0.86 (0.65, 1.14)	0.88 (0.66, 1.17)	0.79 (0.57, 1.10)	0.80 (0.58, 1.12)	1.06 (0.64, 1.76)	1.14 (0.67, 1.93)
PFUA	0.79 (0.59, 1.05)	0.82 (0.61, 1.10)	0.77 (0.55, 1.07)	0.78 (0.56, 1.10)	0.83 (0.50, 1.41)	0.93 (0.54, 1.61)
PFOS	0.88 (0.64, 1.19)	0.86 (0.63, 1.17)	0.79 (0.55, 1.14)	0.77 (0.53, 1.11)	1.12 (0.65, 1.95)	1.13 (0.64, 2.01)
PFHxS	1.16 (0.80, 1.69)	1.16 (0.79, 1.71)	1.05 (0.67, 1.65)	1.03 (0.65, 1.63)	1.45 (0.76, 2.69)	1.58 (0.82, 3.05)
PFHpA	0.96 (0.80, 1.16)	0.95 (0.78, 1.15)	0.93 (0.74, 1.16)	0.91 (0.72, 1.14)	1.04 (0.74, 1.47)	1.06 (0.74, 1.53)
PFBS	1.09 (0.85, 1.39)	1.06 (0.83, 1.37)	0.94 (0.70, 1.26)	0.93 (0.69, 1.25)	1.49 (0.96, 2.32)	1.44 (0.91, 2.26)
PFDoA	0.71 (0.50, 1.01)	0.73 (0.51, 1.04)	0.82 (0.66, 1.01)	0.82 (0.66, 1.03)	0.73 (0.39, 1.35)	0.82 (0.53, 1.58)

*Note*: PFAS concentrations were measured in maternal plasma and have been ln-transformed before entering into the model

^a^ Adjusted for maternal age (years), pre-pregnancy BMI (kg/m^2^), parity (0, ≥ 1), parental educational levels (≤ 12, > 12 years), pregnancy complicating with chronic diseases (no, yes), infant sex (male, female) and gestational age at blood drawn (weeks)

In the stratified analysis, the associations between continuous ln-transformed PFAS and overall PTB, spontaneous and indicated PTB did not differ by infant sex (see Table S3). In the subgroup analyses, the main results remained unchanged when we restricted to women who were nulliparous or without chronic diseases (See Table S4 and S5).

## Discussion

In this prospective study of 2849 mother-infant pairs, we observed no significant associations between maternal PFAS exposure in early pregnancy and the length of gestation, or the risks of overall PTB, spontaneous PTB or indicated PTB at the present exposure levels.

A previous cohort study of 3535 mother-infant pairs in Denmark found that the 3 upper quartiles of PFOA and PFOS in early pregnancy relative to the lowest quartile were significantly associated with PTB. This study also suggested an elevated risk of PTB for per doubling increase of PFHpS and PFDA concentrations [17]. However, this study was based on combined data and samples from three sub-studies. It was, therefore, less certain whether potential biases might have been introduced by the different study designs employed. Another relatively large birth cohort study (*n* = 1645) in Eastern Massachusetts reported that PFOS and PFNA exposures increased the risk of preterm birth [16], but the association was not significant for PFOA or PFHxS. The Taiwan Birth Panel Study included 429 mother-infant pairs to study four PFAS (PFOA, PFOS, PFNA, PFUA) and demonstrated that PFOS level was inversely associated with gestational age at birth (in weeks) and an increased risk of PTB [18]. However, PFAS were measured in cord blood in that study, rendering the causality uncertain.

In contrast to the above studies, we failed to find any association, which was supported by several other studies [19–22]. A Spanish birth cohort study (*n* = 1202) did not observe significant associations of maternal plasma PFOA, PFOS, PFHxS and PFNA in the first trimester with PTB [19]; a cohort study conducted in Canada (*n* = 252) reported that maternal serum PFOA, PFOS and PFHxS in the second trimester did not influence the risk of PTB [21]; the C8 Health Project in the United States measured the risk of PTB in a population with high PFOA exposure, still found no associations of PTB with PFOA or PFOS [22]. Two other studies estimated the prenatal PFOA exposure level through an assessment model and found no increased risk of preterm birth [25, 26]. Likewise, our study showed no associations between PFAS and length of gestation, which was also consistent with these previous studies conducted in Eastern Massachusetts, Spanish and Canada [16, 19, 21].

It should be mentioned that comparing results with previous studies was challenging because these studies were often incomparable in exposure level, study population and exposure window, all of which may affect the effect estimations. First, PFAS exposure levels among these studies varied widely. For instance, the PFOA level ranged from 1.3 ng/mL (geometric mean) [21] to 21.2 ng/mL (median) [20]. Second, exposure backgrounds differed tremendously across these studies as well. For example, the study population in the C8 Health Project lived in a highly PFOA exposed region. Third, divergences also existed in sample type (maternal vs. cord blood) and sampling time (before pregnancy, 1st trimester, 2nd trimester, in labor and after giving birth). It may not be ideal to compare the PFAS effects across studies with different exposure windows, since PFAS levels may vary between non-pregnancy, during pregnancy, and after delivery. Forth, the sample size of these studies varied from 252 [21] to 5262 [20] participants and the PTB rate ranged between 3.2% [17] and 22.5% [20]. Finally, different study populations had different risk profiles. Compared with previous studies, participants in this study were more educated and nulliparous, and few reported smoking or alcohol drinking during pregnancy (see Table S6). Interpretation of our results, therefore, should take into account the background characteristics of our study population.

It is worth noting that none of the previous studies [16–22] differentiated clinical phenotypes of PTB. Since the underlying pathophysiology of PTB differs enormously between spontaneous and indicated PTB [3], it becomes essential to distinguish between these two types of PTB. The ability to assess PFAS exposure during early pregnancy in relation to the risk of subtypes of PTB is a notable strength of our study.

A recurrence risk of PTB could increase by 15 to 50% among women with a previous PTB [3]. On the contrary, birth as an elimination route may lead to lower PFAS blood concentrations [27]. Similarly, PFAS concentrations tend to decrease with higher parity [27], whereas the incidence of PTB appears to increase [28]. As a consequence, potential bias may arise from previous PTB or multiparity. To address this potential effect modification by parity, we repeated our analyses in nulliparous women. We were also able to examine the effects of PFAS in PTB by excluding women with chronic diseases. Our findings consistently suggested no association.

Several limitations of this study are worth noting. First, PTB was reported in one of our previous large-scale studies which conducted in 20 hospitals in Shanghai between 2015 and 2017 with an incidence rate of 5.6% [29]. The present study had a lower incidence of PTB (4.8%), which may be explained by the fact that our study did not include multiple gestation and pregnancies conceived with assisted reproductive technology, the subgroups carrying a substantial risk for PTB [3, 30]. Further, the method of gestational age estimation may influence the rate of PTB. Both the last menstrual period and early ultrasonic measurement were used [31]. Our study was conducted in Shanghai, one of the most advanced cities in China, where the content of prenatal care is comparable to those in western developed countries. Almost all pregnant women receive prenatal care and early ultrasound is performed routinely. The ultrasound measurement has been used to correct the calculated gestational age based on the last menstrual period. When the estimates of gestational age by these two methods differed by more than 2 weeks, the ultrasound estimate was used. Therefore, we believe that the gestational age estimate and PTB rate in our study were reasonably accurate.

Second, we did not adjust for hemodynamics in pregnancy in the analyses. Only two previous studies [16, 19] have adjusted for hemodynamic markers (serum albumin and estimated glomerular filtration rate) in the assessment of associations between PFAS measured in early pregnancy and birth outcomes including PTB. But their results suggested that early pregnancy hemodynamics did not confound the associations. Instead, we adjusted for the gestational week at blood collection to control for the potential hemodynamic difference. The results were virtually the same. Therefore, our findings were less likely to be biased by early pregnancy hemodynamics. Third, while the evaluation of the associations of PFAS exposure with spontaneous and indicated PTB separately was one strength of our study, the sample sizes of the subtypes of PTB were relatively small, which may have led to low precision in our effect estimates. Thus, it is worth noting that failure to detect a significant association does not necessarily mean that there is no effect. Fourth, although we have adjusted for a number of potential confounders, the possibility of residual confounding (e.g., gene, maternal nutrition/dietary factors, and household income) could not be ruled out. Our negative results should be interpreted with caution and need to be confirmed in large-scale studies in other populations.

## Conclusions

Our study found no significant associations between maternal PFAS concentrations in early pregnancy and the length of gestation or the risk of PTB.

## Supplementary information

**Additional file 1: Figure S1**. Associations between PFAS (ng/ml) concentrations and gestational age at birth (GA, weeks). PFAS concentrations were measured in maternal plasma in early pregnancy and have been ln-transformed before entering into the restricted cubic spline regression model. Model adjusted for maternal age, pre-pregnancy BMI, parity, parental educational levels, pregnancy complicating with chronic diseases, infant sex and gestational age at blood drawn.

**Additional file 2: Figure S2**. Associations between non-transformed PFAS (ng/ml) concentrations and gestational age at birth (GA, weeks). PFAS concentrations were measured in maternal plasma in early pregnancy. Model adjusted for maternal age, pre-pregnancy BMI, parity, parental educational levels, pregnancy complicating with chronic diseases, infant sex and gestational age at blood drawn.

**Additional file 3: Figure S3**. Directed acyclic graph (DGA) illustrating confounders and modifier. (A) Path A indicated a direct effect; (B) The associations between PFAS exposure and outcomes of interest were confounded by maternal age, parity, pre-pregnancy BMI, parental educational levels and pregnancy complicating with chronic diseases and gestational age at blood drawn; (C) Fetal gender can be an effect modifier in the associations between PFAS exposure and outcomes of interest.

**Additional file 4: Table S1**. Correlations between plasma concentrations of PFAS. **Table S2**. Associations between ln-transformed plasma concentrations of PFAS in early pregnancy and preterm birth. **Table S3**. Associations between PFAS concentrations in early pregnancy and preterm birth stratified by infant sex. **Table S4**. Associations between PFAS concentrations in early pregnancy and preterm birth in nulliparous women. **Table S5**. Associations between of PFAS concentrations in early pregnancy and preterm birth in women without chronic diseases. **Table S6**. PFAS median levels (ng/mL) in different areas. **Table S7**. Associations between ln-transformed plasma concentrations of PFAS in early pregnancy and length of gestation (weeks). **Table S8**. Linear regression was used to analyzed the associations between plasma concentrations of PFAS in early pregnancy and length of gestation (weeks) in three tertile groups. **Table S9**. Linear regression was used to analyzed the associations between plasma concentrations of PFAS in early pregnancy and preterm birth in three tertile groups. **Table S10**. Associations between PFAS concentrations in early pregnancy and preterm birth with adjustment of education in finer categories (≤12, 12–16, > 16 years). **Table S11**. Associations between PFAS concentrations in early pregnancy and late preterm birth (34–36 weeks).

## Data Availability

The dataset generated and/or analyzed in the current study are not publicly available but can be obtained from the corresponding author on a reasonable request.

## Abbreviations

BMI: Body mass index
CI: Confidence interval
IQRs: Interquartile ranges
LOD: Limit of detection
OR: Odds ratio
PTB: Preterm birth
PPROM: Preterm premature rupture of the membranes
PFAS: Perfluoroalkyl substances
PFOA: Perfluorooctanate
PFOS: Perfluorooctane sulfonate
PFDA: Perfluorodecanoic acid
PFUA: Perfluoroundecanoic acid
PFNA: Perfluorononanoic acid
PFHxS: Perfluorohexanesulfonate
PFHpA: Perfluoroheptanoic acid
PFBS: Perfluorobutane sulfonate
PFDoA: Perfluorododecanoic acid
PFOSA: Perfluorooctane sulfonamide
