# Supplementary figures and images for: Perfluoroalkyl substances exposure in early pregnancy and preterm birth in singleton pregnancies: a prospective cohort study
Source: Environ Health. 2020 Jun 3;19:60. doi: 10.1186/s12940-020-00616-8 (PMC7268357; doi:10.1186/s12940-020-00616-8)

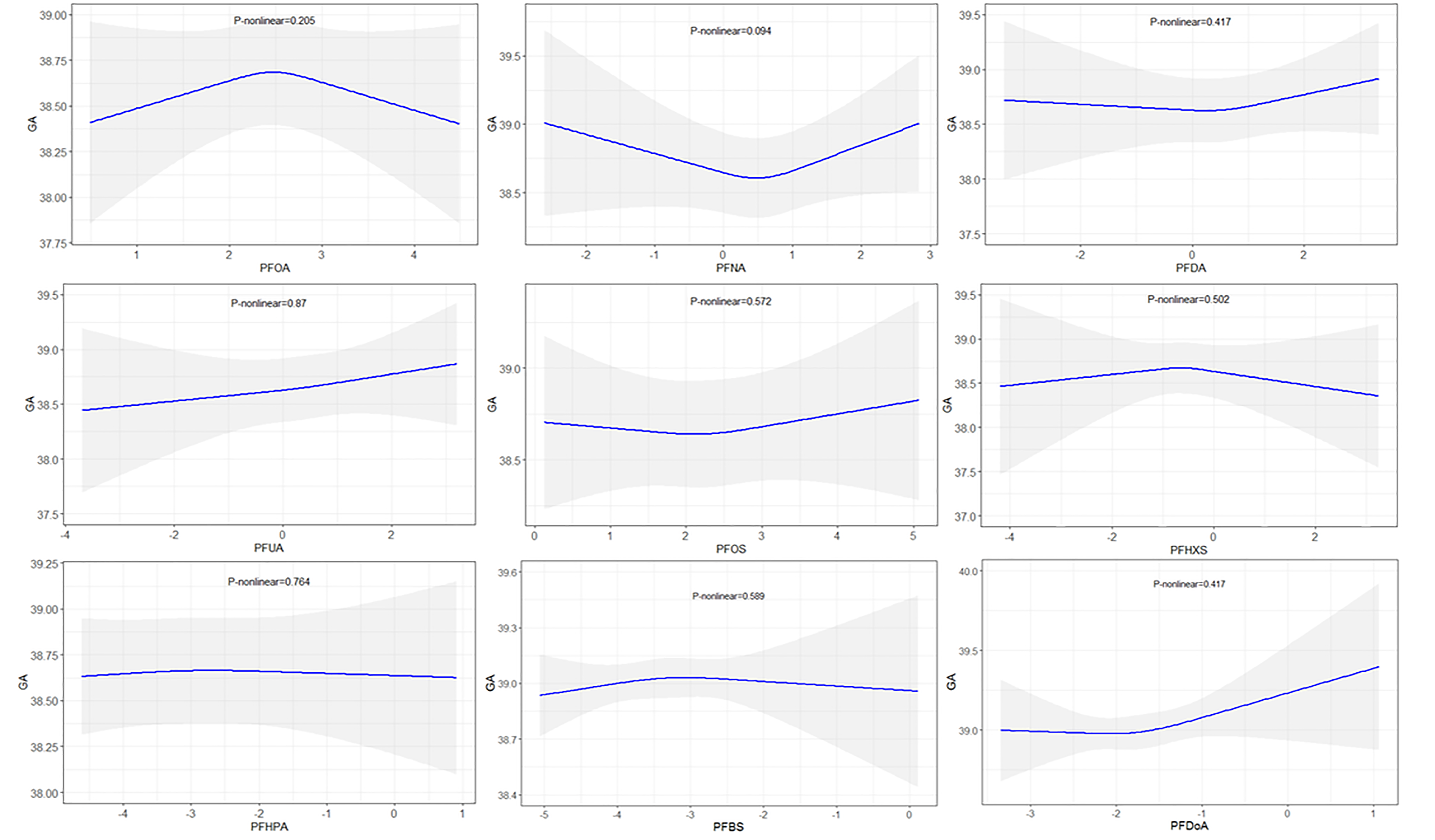

Supplement: Supplementary file 1 — Additional file 1: Figure S1. Associations between PFAS (ng/ml) concentrations and gestational age at birth (GA, weeks). PFAS concentrations were measured in maternal plasma in early pregnancy and have been ln-transformed before entering into the restricted cubic spline regression model. Model adjusted for maternal age, pre-pregnancy BMI, parity, parental educational levels, pregnancy complicating with chronic diseases, infant sex and gestational age at blood drawn. [file 12940_2020_616_MOESM1_ESM.tif]

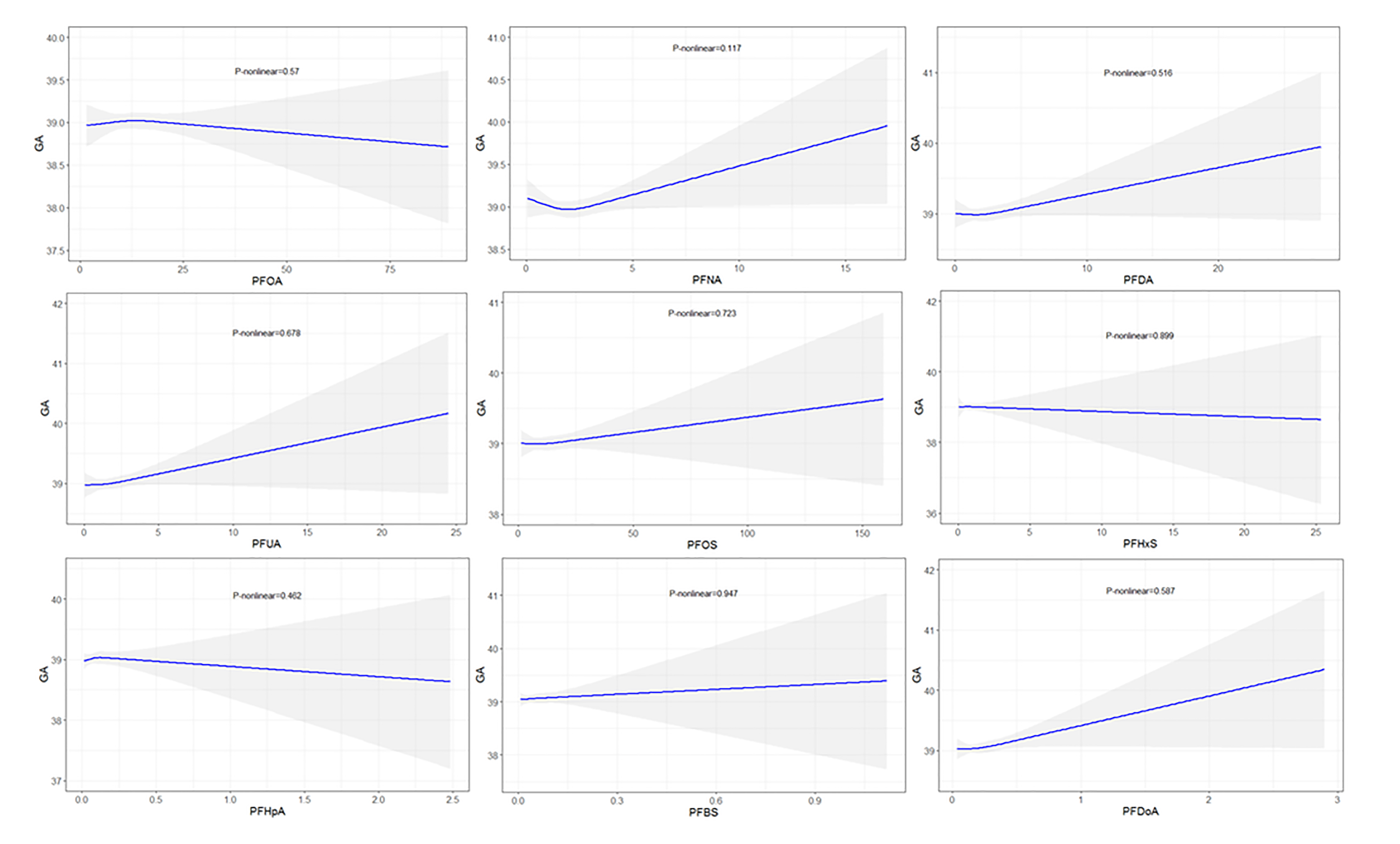

Supplement: Supplementary file 2 — Additional file 2: Figure S2. Associations between non-transformed PFAS (ng/ml) concentrations and gestational age at birth (GA, weeks). PFAS concentrations were measured in maternal plasma in early pregnancy. Model adjusted for maternal age, pre-pregnancy BMI, parity, parental educational levels, pregnancy complicating with chronic diseases, infant sex and gestational age at blood drawn. [file 12940_2020_616_MOESM2_ESM.tif]

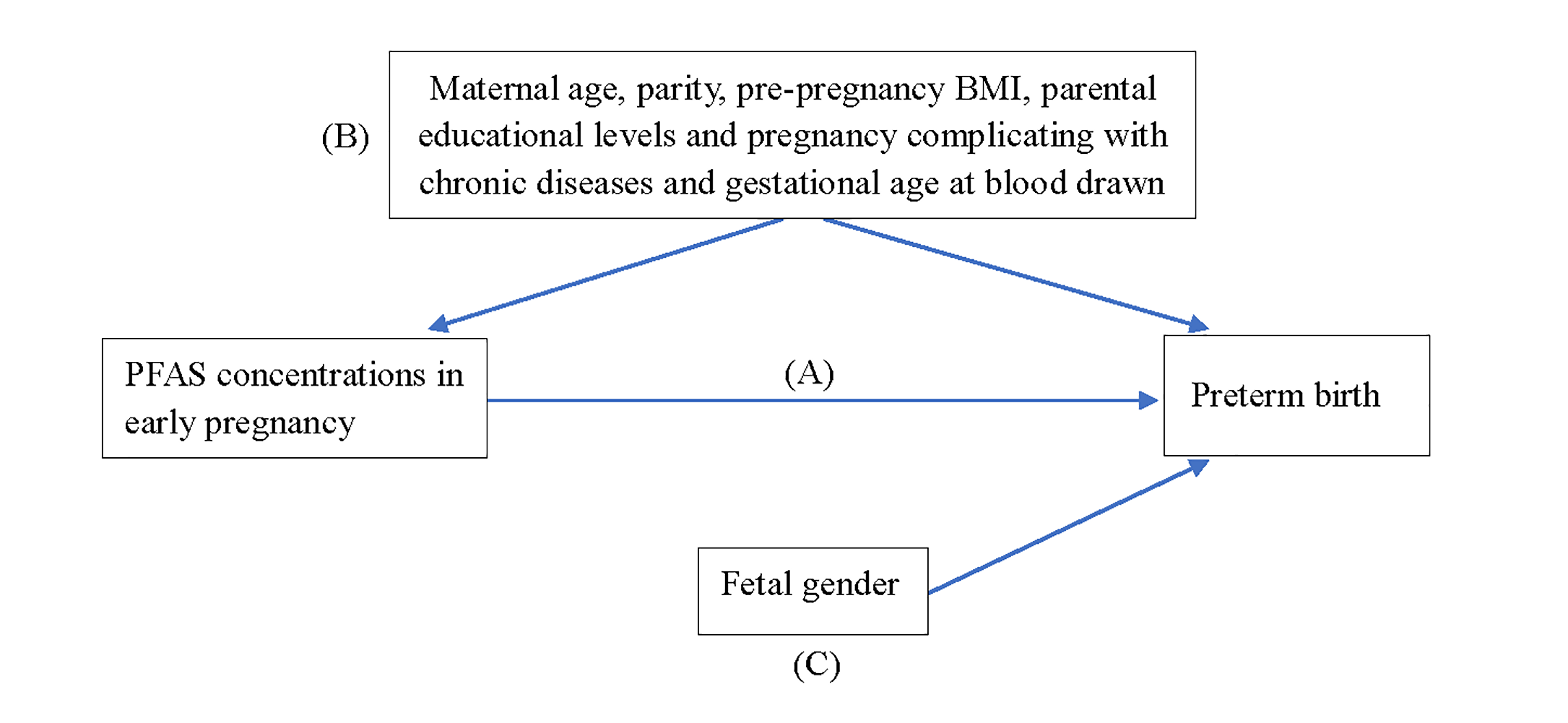

Supplement: Supplementary file 3 — Additional file 3: Figure S3. Directed acyclic graph (DGA) illustrating confounders and modifier. (A) Path A indicated a direct effect; (B) The associations between PFAS exposure and outcomes of interest were confounded by maternal age, parity, pre-pregnancy BMI, parental educational levels and pregnancy complicating with chronic diseases and gestational age at blood drawn; (C) Fetal gender can be an effect modifier in the associations between PFAS exposure and outcomes of interest. [file 12940_2020_616_MOESM3_ESM.tif]
