# Supplementary material for: Perfluoroalkyl substances exposure in early pregnancy and preterm birth in singleton pregnancies: a prospective cohort study
Source: Environ Health. 2020 Jun 3;19:60. doi: 10.1186/s12940-020-00616-8 (PMC7268357; doi:10.1186/s12940-020-00616-8)
Supplement: Supplementary file 4 — Additional file 4: Table S1. Correlations between plasma concentrations of PFAS. Table S2. Associations between ln-transformed plasma concentrations of PFAS in early pregnancy and preterm birth. Table S3. Associations between PFAS concentrations in early pregnancy and preterm birth stratified by infant sex. Table S4. Associations between PFAS concentrations in early pregnancy and preterm birth in nulliparous women. Table S5. Associations between of PFAS concentrations in early pregnancy and preterm birth in women without chronic diseases. Table S6. PFAS median levels (ng/mL) in different areas. Table S7. Associations between ln-transformed plasma concentrations of PFAS in early pregnancy and length of gestation (weeks). Table S8. Linear regression was used to analyzed the associations between plasma concentrations of PFAS in early pregnancy and length of gestation (weeks) in three tertile groups. Table S9. Linear regression was used to analyzed the associations between plasma concentrations of PFAS in early pregnancy and preterm birth in three tertile groups. Table S10. Associations between PFAS concentrations in early pregnancy and preterm birth with adjustment of education in finer categories (≤12, 12–16, > 16 years). Table S11. Associations between PFAS concentrations in early pregnancy and late preterm birth (34–36 weeks). [file 12940_2020_616_MOESM4_ESM.docx]

Table S1. Correlations between plasma concentrations of PFAS.

|  | PFOA | PFNA | PFDA | PFUA | PFOS | PFHxS | PFBS | PFHpA | PFDoA |
| --- | --- | --- | --- | --- | --- | --- | --- | --- | --- |
|  | ρ | ρ | ρ | ρ | ρ | ρ |  |  |  |
| PFOA | 1.00 |  |  |  |  |  |  |  |  |
| PFNA | 0.60 | 1.00 |  |  |  |  |  |  |  |
| PFDA | 0.46 | 0.85 | 1.00 |  |  |  |  |  |  |
| PFUA | 0.40 | 0.82 | 0.94 | 1.00 |  |  |  |  |  |
| PFOS | 0.47 | 0.77 | 0.87 | 0.81 | 1.00 |  |  |  |  |
| PFHxS | 0.55 | 0.46 | 0.40 | 0.35 | 0.43 | 1.00 |  |  |  |
| PFBS | 0.17 | 0.04 | -0.03 | -0.04 | 0.06 | 0.05 | 1.00 |  |  |
| PFHpA | 0.30 | 0.24 | 0.08 | 0.07 | 0.13 | 0.14 | 0.21 | 1.00 |  |
| PFDoA | 0.30 | 0.60 | 0.55 | 0.57 | 0.53 | 0.19 | 0.17 | 0.44 | 1.00 |

Note: ρ: Spearman’s rank correlation coefficient.

Table S2. Associations between ln-transformed plasma concentrations of PFAS in early pregnancy and preterm birth.

|  |  | Overall preterm birth | | Spontaneous preterm birth | | Indicated preterm birth | |
| --- | --- | --- | --- | --- | --- | --- | --- |
| PFAS | Tertiles  (ng/ml) | OR (95% CI) | aOR* (95% CI) | OR (95% CI) | aOR* (95% CI) | OR (95% CI) | aOR* (95% CI) |
| PFOA |  |  |  |  |  |  |  |
|  | T1 | Ref | Ref | Ref | Ref | Ref | Ref |
|  | T2 | 0.77 (0.51, 1.18) | 0.77 (0.50, 1.17) | 0.71 (0.43, 1.16) | 0.71 (0.43, 1.17) | 1.00 (0.46, 2.17) | 0.96 (0.44, 2.11) |
|  | T3 | 0.82 (0.54, 1.24) | 0.81 (0.54, 1.24) | 0.76 (0.47, 1.23) | 0.76 (0.46, 1.22) | 1.00 (0.46, 2.17) | 1.02 (0.47, 2.22) |
| PFNA |  |  |  |  |  |  |  |
|  | T1 | Ref | Ref | Ref | Ref | Ref | Ref |
|  | T2 | 0.91 (0.60, 1.38) | 0.91 (0.60, 1.38) | 0.97 (0.60, 1.58) | 0.97 (0.59, 1.57) | 0.80 (0.37, 1.72) | 0.82 (0.38, 1.77) |
|  | T3 | 0.85 (0.56, 1.29) | 0.88 (0.57, 1.34) | 0.88 (0.53, 1.45) | 0.90 (0.54, 1.50) | 0.80 (0.37, 1.71) | 0.86 (0.39, 1.87) |
| PFDA |  |  |  |  |  |  |  |
|  | T1 | Ref | Ref | Ref | Ref | Ref | Ref |
|  | T2 | 1.04 (0.69, 1.57) | 1.05 (0.70, 1.59) | 1.04 (0.65, 1.67) | 1.05 (0.65, 1.69) | 1.08 (0.49, 2.38) | 1.11 (0.50, 2.48) |
|  | T3 | 0.85 (0.55, 1.30) | 0.87 (0.56, 1.35) | 0.76 (0.45, 1.26) | 0.76 (0.45, 1.29) | 1.17 (0.54, 2.54) | 1.30 (0.59, 2.89) |
| PFUA |  |  |  |  |  |  |  |
|  | T1 | Ref | Ref | Ref | Ref | Ref | Ref |
|  | T2 | 0.86 (0.57, 1.29) | 0.87 (0.58, 1.32) | 0.89 (0.55, 1.43) | 0.90 (0.56, 1.45) | 0.78 (0.35, 1.73) | 0.84 (0.38, 1.88) |
|  | T3 | 0.79 (0.52, 1.21) | 0.86 (0.56, 1.32) | 0.72 (0.44, 1.19) | 0.75 (0.45, 1.25) | 1.00 (0.47, 2.11) | 1.23 (0.57, 2.67) |
| PFOS |  |  |  |  |  |  |  |
|  | T1 | Ref | Ref | Ref | Ref | Ref | Ref |
|  | T2 | 0.62 (0.40, 0.94) | 0.61 (0.40, 0.94) | 0.60 (0.35, 0.94) | 0.56 (0.34, 0.94) | 0.78 (0.35, 1.73) | 0.79 (0.35, 1.78) |
|  | T3 | 0.74 (0.50, 1.12) | 0.73 (0.48, 1.10) | 0.66 (0.41, 1.07) | 0.65 (0.40, 1.05) | 1.00 (0.47, 2.10) | 0.99 (0.46, 2.12) |
| PFHxS |  |  |  |  |  |  |  |
|  | T1 | Ref | Ref | Ref | Ref | Ref | Ref |
|  | T2 | 1.07 (0.70, 1.65) | 1.07 (0.70, 1.65) | 1.14 (0.69, 1.87) | 1.14 (0.69, 1.87) | 0.92 (0.40, 2.09) | 0.93 (0.40, 2.13) |
|  | T3 | 1.17 (0.77, 1.79) | 1.17 (0.77, 1.79) | 1.10 (0.67, 1.82) | 1.10 (0.66, 1.82) | 1.34 (0.63, 2.84) | 1.43 (0.66, 3.08) |
| PFHpA |  |  |  |  |  |  |  |
|  | T1 | Ref | Ref | Ref | Ref | Ref | Ref |
|  | T2 | 0.83 (0.55, 1.27) | 0.83 (0.54, 1.27) | 0.69 (0.42, 1.15) | 0.70 (0.42, 1.17) | 1.23 (0.59, 2.58) | 1.17 (0.55, 2.48) |
|  | T3 | 0.87 (0.58, 1.32) | 0.87 (0.56, 1.33) | 0.91 (0.57, 1.47) | 0.91 (0.56, 1.49) | 0.77 (0.33, 1.75) | 0.77 (0.33, 1.82) |
| PFBS |  |  |  |  |  |  |  |
|  | T1 | Ref | Ref | Ref | Ref | Ref | Ref |
|  | T2 | 0.82 (0.50, 1.36) | 0.80 (0.48, 1.32) | 0.85 (0.48, 1.49) | 0.83 (0.47, 1.46) | 0.75 (0.26, 2.17) | 0.72 (0.25, 2.10) |
|  | T3 | 1.27 (0.81, 2.00) | 1.23 (0.77, 1.95) | 0.92 (0.53, 1.60) | 0.90 (0.52, 1.58) | 2.40 (1.05, 5.53) | 2.23 (0.96, 5.19) |
| PFDoA |  |  |  |  |  |  |  |
|  | T1 | Ref | Ref | Ref | Ref | Ref | Ref |
|  | T2 | 0.74 (0.37, 1.18) | 0.76 (0.48, 1.21) | 0.71 (0.41, 1.22) | 0.73 (0.42, 1.26) | 0.84 (0.69, 1.90) | 0.86 (0.38, 1.95) |
|  | T3 | 0.63 (0.39, 1.02) | 0.64 (0.39, 1.05) | 0.61 (0.35, 1.08) | 0.57 (0.32, 1.03) | 0.69 (0.29, 1.62) | 0.83 (0.34, 2.01) |

*Adjusted for maternal age (years), pre-pregnancy BMI (kg/m^2^), parity (0, ≥1, unknown), parental educational levels (≤12, >12 years), pregnancy complicating with chronic diseases (no, yes), infant sex (male, female) and gestational age at blood drawn (weeks).

Table S3. Associations between PFAS concentrations in early pregnancy and preterm birth stratified by infant sex.

| PFAS (ng/ml) | Overall preterm birth | | | | Spontaneous preterm birth | | Non-spontaneous preterm birth | | |
| --- | --- | --- | --- | --- | --- | --- | --- | --- | --- |
| Male | N_preterm_=75/ N_term_ =1381 | | | | N_preterm_=53/ N_term_ =1381 | | N_preterm_=22/ N_term_ =1381 | | |
|  | OR (95% CI) | aOR* (95% CI) | | OR (95% CI) | | aOR* (95% CI) | OR (95% CI) | | aOR* (95% CI) |
| PFOA | 1.00 (0.58, 1.74) | 1.02 (0.59, 1.78) | | 0.92 (0.48, 1.76) | | 0.95 (0.49, 1.81) | 1.23 (0.46, 3.31) | | 1.23 (0.44, 3.39) |
| PFNA | 1.00 (0.65, 1.55) | 0.99 (0.63, 1.54) | | 0.99 (0.59, 1.66) | | 0.98 (0.58, 1.65) | 1.04 (0.47, 2.28) | | 1.02 (0.45, 2.34) |
| PFDA | 0.91 (0.63, 1.33) | 0.87 (0.60, 1.28) | | 0.87 (0.56, 1.36) | | 0.84 (0.54, 1.31) | 1.01 (0.51, 1.98) | | 0.98 (0.49, 1.98) |
| PFUA | 0.85 (0.58, 1.25) | 0.83 (0.56, 1.22) | | 0.83 (0.53, 1.29) | | 0.79 (0.50, 1.24) | 0.92 (0.46, 1.82) | | 0.94 (0.45, 1.92) |
| PFOS | 0.99 (0.66, 1.48) | 0.94 (0.62, 1.41) | | 0.96 (0.60, 1.55) | | 0.93 (0.57, 1.50) | 1.07 (0.52, 2.20) | | 0.98 (0.46, 2.09) |
| PFHxS | 1.00 (0.58, 1.72) | 1.00 (0.58, 1.74) | | 1.08 (0.58, 2.04) | | 1.09 (0.58, 2.05) | 0.81 (0.29, 2.2) | | 0.81 (0.28, 2.3) |
| PFHpA | 1.07 (0.80, 1.42) | 1.08 (0.81, 1.45) | | 1.03 (0.74, 1.45) | | 1.05 (0.74, 1.48) | 1.15 (0.69, 1.91) | | 1.18 (0.70, 1.99) |
| PFBS | 1.25 (0.88, 1.77) | 1.23 (0.86, 1.76) | | 1.11 (0.73, 1.68) | | 1.10 (0.72, 1.67) | 1.65 (0.89, 3.06) | | 1.57 (0.82, 3.01) |
| PFDoA | 0.87 (0.54, 1.40) | 0.81 (0.50, 1.33) | | 0.78 (0.44, 1.39) | | 0.71 (0.40, 1.28) | 1.09 (0.48, 2.46) | | 1.13 (0.47, 2.68) |
| Female | N_preterm_=61/ N_term_ =1332 | | N_preterm_=44/ N_term_ =1332 | | | | | N_preterm_=17/ N_term_ =1332 | |
|  | OR (95% CI) | aOR* (95% CI) | | OR (95% CI) | | aOR* (95% CI) | OR (95% CI) | | aOR* (95% CI) |
| PFOA | 0.83 (0.44, 1.53) | 0.82 (0.44, 1.55) | | 0.55 (0.27, 1.13) | | 0.54 (0.26, 1.13) | 2.28 (0.76, 6.77) | | 2.64 (0.83, 8.39) |
| PFNA | 0.66 (0.40, 1.10) | 0.74 (0.44, 1.25) | | 0.61 (0.34, 1.12) | | 0.68 (0.37, 1.25) | 0.80 (0.31, 2.05) | | 0.97 (0.36, 2.61) |
| PFDA | 0.79 (0.52, 1.20) | 0.88 (0.57, 1.35) | | 0.69 (0.42, 1.12) | | 0.75 (0.45, 1.24) | 1.10 (0.51, 2.4) | | 1.38 (0.61, 3.11) |
| PFUA | 0.69 (0.44, 1.08) | 0.81 (0.51, 1.28) | | 0.68 (0.40, 1.15) | | 0.77 (0.45, 1.31) | 0.71 (0.31, 1.63) | | 0.93 (0.38, 2.25) |
| PFOS | 0.72 (0.44, 1.16) | 0.74 (0.45, 1.22) | | 0.59 (0.33, 1.03) | | 0.59 (0.33, 1.06) | 1.18 (0.50, 2.78) | | 1.35 (0.56, 3.26) |
| PFHxS | 1.34 (0.79, 2.25) | 1.33 (0.77, 2.27) | | 1.00 (0.52, 1.94) | | 0.96 (0.49, 1.89) | 2.22 (1.06, 4.65) | | 2.56 (1.18, 5.53) |
| PFHpA | 0.84 (0.61, 1.16) | 0.88 (0.57, 1.12) | | 0.81 (0.56, 1.18) | | 0.77 (0.52, 1.15) | 0.93 (0.51, 1.69) | | 0.88 (0.47, 1.64) |
| PFBS | 0.93 (0.65, 1.34) | 0.90 (0.63, 1.28) | | 0.79 (0.52, 1.22) | | 0.77 (0.50, 1.18) | 1.35 (0.72, 2.52) | | 1.25 (0.66, 2.37) |
| PFDoA | 0.81 (0.50, 1.33) | 0.63 (0.37, 1.07) | | 0.64 (0.35, 1.17) | | 0.67 (0.36, 1.23) | 0.44 (0.17, 1.15) | | 0.53 (0.19, 1.47) |

Note: PFAS concentrations were measured in maternal plasma and have been ln-transformed before entering into the model.* Adjusted for maternal age (years), pre-pregnancy BMI (kg/m^2^), parity (0, ≥1), parental educational levels (≤12, >12 years), pregnancy complicating with chronic diseases (no, yes) and gestational age at blood drawn (weeks).

Table S4. Associations between PFAS concentrations in early pregnancy and preterm birth in nulliparous women.

| PFAS (ng/ml) | Overall preterm birth | | Spontaneous preterm birth | | Indicated preterm birth | |
| --- | --- | --- | --- | --- | --- | --- |
|  | N_preterm_=113/ N_term_ =2322 | | N_preterm_=82/ _Nterm_ =2322 | | N_preterm_=31/ N_term_ =2322 | |
|  | OR (95% CI) | aOR* (95% CI) | OR (95% CI) | aOR* (95% CI) | OR (95% CI) | aOR* (95% CI) |
| PFOA | 1.10 (0.70, 1.72) | 1.09 (0.69, 1.71) | 0.88 (0.52, 1.49) | 0.88 (0.52, 1.49) | 1.95 (0.86, 4.41) | 1.96 (0.84, 4.57) |
| PFNA | 0.90 (0.63, 1.29) | 0.90 (0.62, 1.30) | 0.84 (0.55, 1.28) | 0.84 (0.55, 1.29) | 1.08 (0.55, 2.12) | 1.10 (0.55, 2.23) |
| PFDA | 0.88 (0.64, 1.20) | 0.90 (0.66, 1.23) | 0.79 (0.55, 1.14) | 0.80 (0.56, 1.15) | 1.14 (0.64, 2.03) | 1.23 (0.68, 2.25) |
| PFUA | 0.79 (0.58, 1.09) | 0.83 (0.60, 1.14) | 0.76 (0.53, 1.10) | 0.77 (0.53, 1.12) | 0.89 (0.49, 1.61) | 0.99 (0.53, 1.85) |
| PFOS | 0.92 (0.66, 1.30) | 0.90 (0.64, 1.28) | 0.80 (0.54, 1.20) | 0.78 (0.52, 1.17) | 1.32 (0.71, 2.45) | 1.31 (0.69, 2.49) |
| PFHxS | 1.26 (0.83, 1.90) | 1.23 (0.80, 1.88) | 1.13 (0.69, 1.85) | 1.09 (0.66, 1.79) | 1.62 (0.79, 3.34) | 1.69 (0.78, 3.62) |
| PFHpA | 1.01 (0.80, 1.27) | 0.97 (0.76, 1.24) | 0.96 (0.74, 1.27) | 0.93 (0.70, 1.24) | 1.14 (0.74, 1.75) | 1.08 (0.69, 1.70) |
| PFBS | 1.17 (0.89, 1.52) | 1.13 (0.86, 1.47) | 1.03 (0.76, 1.42) | 1.01 (0.74, 1.38) | 1.56 (0.96, 2.51) | 1.49 (0.91, 2.45) |
| PFDoA | 0.75 (0.52, 1.09) | 0.74 (0.50, 1.07) | 0.74 (0.48, 1.15) | 0.70 (0.45, 1.08) | 0.78 (0.40, 1.54) | 0.85 (0.43, 1.71) |

Note: PFAS concentrations were measured in maternal plasma and have been ln-transformed before entering into the model.

* Adjusted for maternal age (years), pre-pregnancy BMI (kg/m^2^), parental educational levels (≤12, >12 years), pregnancy complicating with chronic diseases (no, yes), infant sex (male, female) and gestational age at blood drawn (weeks).

Table S5. Associations between of PFAS concentrations in early pregnancy and preterm birth in women without chronic diseases.

| PFAS (ng/ml) | Overall preterm birth | | Spontaneous preterm birth | | Indicated preterm birth | |
| --- | --- | --- | --- | --- | --- | --- |
|  | N_preterm_=125/ N_term_ =2528 | | N_preterm_=87/ N_term_ =2528 | | N_preterm_=38/ N_term_ =2528 | |
|  | OR (95% CI) | aOR* (95% CI) | OR (95% CI) | aOR* (95% CI) | OR (95% CI) | aOR* (95% CI) |
| PFOA | 1.01 (0.66, 1.55) | 0.99 (0.64, 1.53) | 0.79 (0.47, 1.32) | 0.78 (0.47, 1.30) | 1.76 (0.83, 3.73) | 1.80 (0.83, 3.88) |
| PFNA | 0.85 (0.60, 1.19) | 0.85 (0.60 1.20) | 0.82 (0.55, 1.24) | 0.82 (0.54, 1.24) | 0.91 (0.50, 1.67) | 0.96 (0.51, 1.80) |
| PFDA | 0.85 (0.63, 1.14) | 0.85 (0.63, 1.14) | 0.78 (0.58, 1.11) | 0.77 (0.55, 1.10) | 1.01 (0.60, 1.69) | 1.09 (0.64, 1.86) |
| PFUA | 0.79 (0.59, 1.07) | 0.81 (0.59, 1.10) | 0.79 (0.55, 1.13) | 0.78 (0.54, 1.12) | 0.79 (0.47, 1.35) | 0.88 (0.51, 1.55) |
| PFOS | 0.85 (0.62, 1.18) | 0.82 (0.59, 1.13) | 0.76 (0.52, 1.12) | 0.73 (0.49, 1.07) | 1.10 (0.63, 1.93) | 1.11 (0.62, 1.97) |
| PFHxS | 1.23 (0.83, 1.82) | 1.21 (0.81, 1.81) | 1.07 (0.66, 1.75) | 1.05 (0.65, 1.72) | 1.59 (0.84, 3.03) | 1.66 (0.86, 3.18) |
| PFHpA | 0.98 (0.79, 1.23) | 0.97 (0.77, 1.23) | 0.97 (0.74, 1.26) | 0.96 (0.73, 1.27) | 1.02 (0.68, 1.51) | 1.00 (0.66, 1.51) |
| PFBS | 1.10 (0.85, 1.42) | 1.06 (0.82, 1.38) | 0.94 (0.69, 1.29) | 0.92 (0.67, 1.26) | 1.48 (0.95, 2.30) | 1.43 (0.91, 2.25) |
| PFDoA | 0.78 (0.54, 1.12) | 0.78 (0.54, 1.13) | 0.80 (0.52, 1.25) | 0.77 (0.49, 1.19) | 0.72 (0.38, 1.35) | 0.81 (0.42, 1.56) |

Note: PFAS concentrations were measured in maternal plasma and have been ln-transformed before entering into the model.

*Adjusted for maternal age (years), pre-pregnancy BMI (kg/m^2^), parity (0, ≥1), parental educational levels (≤12, >12 years), pregnancy complicating with chronic diseases (no, yes), infant sex (male, female) and gestational age at blood drawn (weeks).

Table S6. PFAS median levels (ng/mL) in different areas.

|  |  |  |  |  |  | PFAS | | | | | | | | | |
| --- | --- | --- | --- | --- | --- | --- | --- | --- | --- | --- | --- | --- | --- | --- | --- |
| Location | Study period | PFAS matrix | N | PTB: n (%) | Maternal characteristics | PFOA | PFNA | PFDA | PFUA | PFOS | PFHxS | PFHpA | PFBS | PFDoA | PFOSA |
| China  (Shanghai)* | 2013-2016 | Maternal plasma (1^st^ T) | 2849 | 136 (4.8) | Age < 35 y: 93.3%  Normal BMI: 74.4%  Smoking during early pregnancy: 0.3%  Education > 12 y: 95.0%  Nulliparous：85.5% | 11.85 | 1.69 | 1.69 | 1.39 | 9.33 | 0.54 | 0.06 | 0.04 | 0.16 | # |
| U.S.[1] | 2000-2006 | Maternal serum/  (five year after delivery) | 1845 (PFOA)  5262  (PFOS) | 329 (20.9) for PFOA;  1015 (22.5) for PFOS | Age < 35 y: 88.9% for PFOA and 91.1% for PFOS  BMI: not shown  Current or former smoking: 50.4% and 54.1%  Education > 12 y: 58.4% and 58.2%  Nulliparous: 45.4% and 48.0% | 21.2 | N/A | N/A | N/A | 13.6 | N/A | N/A | N/A | N/A | N/A |
| U.S.[2] | 2005-2010 | Maternal serum (before or during pregnancy) | 1630 | 158 (9.7) | Age < 30 y: 68%  Normal BMI: 41%  Current or former smoking: 52%  Education > 12 y: 68%  Nulliparous: 35% | 14.3 | N/A | N/A | N/A | 13.9 | N/A | N/A | N/A | N/A | N/A |
| Spain[3] | 2003-2008 | Maternal plasma (1^st^ T) | 1202 | 45  (4) | Mean age: 30.7 y  Mean BMI: 23.6 kg/m^2^  Smoker: not shown  Education: university (35%)  Nulliparous: 56% | 2.35 | 0.66 | N/A | N/A | 6.05 | 0.58 | N/A | N/A | N/A | N/A |
| Canada[4] | 2005-2006 | Maternal plasma (2^nd^ T) | 252 | 21 (8.3) | Age < 35 y: 83.0%  BMI: not shown  Ever smoked during pregnancy: 11.1%  Education: not show  Parity: not show | 1.3^&^ | N/A | N/A | N/A | 7.4^&^ | 1.1^&^ | N/A | N/A | N/A | N/A |
| Denmark[5] | 1996-2002 | Maternal plasma (1^st^ and 2^nd^ T) and umbilical cord blood plasma | 3535 | 112 (3.2) | Age < 35 y: 83.7%  Normal BMI: 68.0%  Education: not shown  Smoking during pregnancy: 28.3%  Nulliparous: 47.1% | 4.6 | 0.5 | 0.2 | N/A | 30.1 | 1.0 | N/A | N/A | N/A | N/A |
| US[6] | 1999-2002 | Maternal plasma (early pregnancy) | 1645 | 120 (7.3) | Age < 35 y: 72.2%  Normal BMI: 58.0%  Education: college or Graduate degree (64.5%)  Former or current smoking: 32.0%  Nulliparous: 48.6% | 5.8 | 0.7 | N/A | N/A | 25.7 | 2.4 | N/A | N/A | N/A | N/A |
| China  (Taiwan) [7] | 2004-2005 | Umbilical Cord Blood | 429 | 40 (9.3) | Age < 35 y: 77.2%  Normal BMI: 78.8%  Education > 12 y: 45.0%  Smoker: 0  Nulliparous: 48.3% | 1.84^&^ | 2.36^&^ | N/A | 10.26^&^ | 5.94^&^ | N/A | N/A | N/A | N/A | N/A |

* our study; T: trimester; N/A: The contaminant was not detected; #: The detection rate is extremely low; &: Geometric mean.

Reference for Table S6.

1. Stein CR, Savitz DA, Dougan M: **Serum levels of perfluorooctanoic acid and perfluorooctane sulfonate and pregnancy outcome**. *Am J Epidemiol* 2009, **170**(7):837-846.

2. Darrow LA, Stein CR, Steenland K: **Serum perfluorooctanoic acid and perfluorooctane sulfonate concentrations in relation to birth outcomes in the Mid-Ohio Valley, 2005-2010**. *Environ Health Perspect* 2013, **121**(10):1207-1213.

3. Manzano-Salgado CB, Casas M, Lopez-Espinosa MJ, Ballester F, Iniguez C, Martinez D, Costa O, Santa-Marina L, Pereda-Pereda E, Schettgen T *et al*: **Prenatal exposure to perfluoroalkyl substances and birth outcomes in a Spanish birth cohort**. *Environ Int* 2017, **108**:278-284.

4. Hamm MP, Cherry NM, Chan E, Martin JW, Burstyn I: **Maternal exposure to perfluorinated acids and fetal growth**. *J Expo Sci Environ Epidemiol* 2010, **20**(7):589-597.

5. Meng Q, Inoue K, Ritz B, Olsen J, Liew Z: **Prenatal Exposure to Perfluoroalkyl Substances and Birth Outcomes; An Updated Analysis from the Danish National Birth Cohort**. *Int J Environ Res Public Health* 2018, **15**(9).

6. Sagiv SK, Rifas-Shiman SL, Fleisch AF, Webster TF, Calafat AM, Ye X, Gillman MW, Oken E: **Early-Pregnancy Plasma Concentrations of Perfluoroalkyl Substances and Birth Outcomes in Project Viva: Confounded by Pregnancy Hemodynamics?** *Am J Epidemiol* 2018, **187**(4):793-802.

7. Chen MH, Ha EH, Wen TW, Su YN, Lien GW, Chen CY, Chen PC, Hsieh WS: **Perfluorinated compounds in umbilical cord blood and adverse birth outcomes**. *PLoS One* 2012, **7**(8):e42474.

Table S7. Associations between ln-transformed plasma concentrations of PFAS in early pregnancy and length of gestation (weeks).

|  |  | Gestational age | |
| --- | --- | --- | --- |
| PFAS | Tertiles (ng/ml) | OR (95% CI) | aOR* (95% CI) |
| PFOA |  |  |  |
|  | T1 | Ref | Ref |
|  | T2 | 0.11 (-0.02, 0.25) | 0.11 (-0.03, 0.24) |
|  | T3 | 0.00 (-0.13, 0.14) | -0.01 (-0.15, 0.12) |
| PFNA |  |  |  |
|  | T1 | Ref | Ref |
|  | T2 | 0.002 (-0.13, 0.14) | 0.01 (-0.13, 0.14) |
|  | T3 | 0.03 (-0.11, 0.16) | 0.01 (-0.12, 0.15) |
| PFDA |  |  |  |
|  | T1 | Ref | Ref |
|  | T2 | -0.01 (-0.15, 0.13) | -0.00 (-0.14, 0.13) |
|  | T3 | 0.03 (-0.11, 0.16) | 0.03 (-0.10, 0.17) |
| PFUA |  |  |  |
|  | T1 | Ref | Ref |
|  | T2 | 0.07 (-0.06, 0.21) | 0.07 (-0.06, 0.21) |
|  | T3 | 0.07 (-0.06, 0.21) | 0.04 (-0.10, 0.17) |
| PFOS |  |  |  |
|  | T1 | Ref | Ref |
|  | T2 | 0.06 (-0.08, 0.20) | 0.08 (-0.06, 0.21) |
|  | T3 | 0.02 (-0.12, 0.16) | 0.06 (-0.08, 0.19) |
| PFHxS |  |  |  |
|  | T1 | Ref | Ref |
|  | T2 | 0.04 (-0.10, 0.17) | 0.05 (-0.09, 0.18) |
|  | T3 | 0.01 (-0.13, 0.15) | 0.00 (-0.13, 0.14) |
| PFHpA |  |  |  |
|  | T1 | Ref | Ref |
|  | T2 | 0.02 (-0.12, 0.16) | 0.01 (-0.13, 0.14) |
|  | T3 | 0.05 (-0.08, 0.19) | 0.04 (-0.10, 0.17) |
| PFBS |  |  |  |
|  | T1 | Ref | Ref |
|  | T2 | 0.07 (0.08, 0.21) | 0.08 (-0.06, 0.23) |
|  | T3 | -0.03 (-0.17, 0.12) | 0.02 (-0.12, 0.17) |
| PFDoA |  |  |  |
|  | T1 | Ref | Ref |
|  | T2 | 0.04 (-0.10, 0.19) | 0.03 (-0.11, 0.17) |
|  | T3 | 0.12 (-0.02, 0.27) | 0.10 (-0.05, 0.25) |

*Adjusted for maternal age (years), pre-pregnancy BMI (kg/m^2^), parity (0, ≥1, unknown), parental educational levels (≤12, >12 years), pregnancy complicating with chronic diseases (no, yes), infant sex (male, female) and gestational age at blood drawn (weeks).

Table S8. Linear regression was used to analyzed the associations between plasma concentrations of PFAS in early pregnancy and length of gestation (weeks) in three tertile groups.

|  |  | Gestational age | |
| --- | --- | --- | --- |
| PFAS (ln-ng/mL) | Tertiles | β (95% CI) | adjusted β* (95% CI) |
| PFOA |  |  |  |
|  | T1 | 0.09 (-0.34, 0.51) | 0.11 (-0.31, 0.54) |
|  | T2 | -0.68 (-1.75, 0.39) | -0.69 (-1.75, 0.37) |
|  | T3 | 0.03 (-0.29, 0.34) | 0.03 (-0.29, 0.35) |
| PFNA |  |  |  |
|  | T1 | -0.24 (-0.56, 0.07) | -0.18 (-0.50, 0.13) |
|  | T2 | 0.23 (-0.63, 1.09) | 0.18 (-0.67, 1.03) |
|  | T3 | 0.29 (0.01, 0.57) | 0.22 (-0.06, 0.51) |
| PFDA |  |  |  |
|  | T1 | -0.04 (-0.32, 0.23) | -0.003 (-0.27, 0.27) |
|  | T2 | 0.42 (-0.26, 1.10) | 0.34 (-0.33, 1.01) |
|  | T3 | 0.10 (-0.15, 0.35) | 0.12 (-0.13, 0.37) |
| PFUA |  |  |  |
|  | T1 | -0.004 (-0.28, 0.27) | 0.03 (-0.24, 0.31) |
|  | T2 | 0.37 (-0.35, 1.08) | 0.40 (-0.31, 1.11) |
|  | T3 | 0.21 (-0.07, 0.49) | 0.22 (-0.07, 0.50) |
| PFOS |  |  |  |
|  | T1 | -0.38 (-0.73, -0.03) | -0.27 (-0.62, 0.08) |
|  | T2 | 0.08 (-0.64, 0.79) | 0.26 (-0.43, 0.96) |
|  | T3 | 0.0003 (-0.26, 0.26) | 0.03 (-0.24, 0.29) |
| PFHxS |  |  |  |
|  | T1 | -0.19 (-0.68, 0.29) | -0.04 (-0.52, 0.44) |
|  | T2 | 0.08 (-1.01, 1.17) | 0.14 (-0.94, 1.22) |
|  | T3 | -0.07 (-0.31, 0.17) | -0.06 (-0.31, 0.18) |
| PFHpA |  |  |  |
|  | T1 | -0.01 (-0.23, 0.20) | 0.01 (-0.22, 0.21) |
|  | T2 | 0.37 (-0.25, 0.98) | 0.37 (-0.24, 0.98) |
|  | T3 | -0.07 (-0.26, 0.13) | -0.06 (-0.26, 0.13) |
| PFBS |  |  |  |
|  | T1 | 0.06 (-0.24, 0.37) | 0.09 (-0.22, 0.39) |
|  | T2 | -0.25 (-0.75, 0.25) | -0.21 (-0.70, 0.29) |
|  | T3 | 0.06 (-0.22, 0.34) | 0.10 (-0.18, 0.38) |
| PFDoA |  |  |  |
|  | T1 | 0.02 (-0.53, 0.58) | -0.01 (-0.56, 0.54) |
|  | T2 | -0.15 (-0.78, 0.48) | -0.18 (-0.82, 0.46) |
|  | T3 | 0.16 (-0.09, 0.40) | 0.15 (-0.09, 0.39) |

*Adjusted for maternal age (years), pre-pregnancy BMI (kg/m^2^), parity (0, ≥1, unknown), parental educational levels (≤12, >12 years), pregnancy complicating with chronic diseases (no, yes), infant sex (male, female) and gestational age at blood drawn (weeks).

Table S9. Linear regression was used to analyzed the associations between plasma concentrations of PFAS in early pregnancy and preterm birth in three tertile groups.

|  |  | Overall preterm birth | |
| --- | --- | --- | --- |
| PFAS (ln-ng/mL) | Tertiles | OR (95% CI) | aOR* (95% CI) |
| PFOA |  |  |  |
|  | T1 | 0.88 (0.28, 2.73) | 0.81 (0.26, 2.53) |
|  | T2 | 2.54 (0.09, 69.45) | 2.66 (0.10, 73.96) |
|  | T3 | 1.98 (0.74, 5.34) | 2.05 (0.75, 5.63) |
| PFNA |  |  |  |
|  | T1 | 0.93 (0.36, 2.41) | 0.85 (0.32, 2.30) |
|  | T2 | 1.90 (0.16, 22.31) | 2.33 (0.19, 29.09) |
|  | T3 | 0.57 (0.19, 1.70) | 0.69 (0.23, 2.07) |
| PFDA |  |  |  |
|  | T1 | 0.86 (0.38, 1.92) | 0.82 (0.36, 1.87) |
|  | T2 | 0.58 (0.08, 3.97) | 0.66 (0.09, 4.69) |
|  | T3 | 0.78 (0.31, 1.96) | 0.84 (0.33, 2.12) |
| PFUA |  |  |  |
|  | T1 | 0.95 (0.43, 2.11) | 0.95 (0.42, 2.15) |
|  | T2 | 0.18 (0.02, 1.53) | 0.15 (0.02, 1.39) |
|  | T3 | 0.41 (0.13, 1.33) | 0.47 (0.15, 1.55) |
| PFOS |  |  |  |
|  | T1 | 3.03 (0.98, 9.38) | 2.67 (0.85, 8.29) |
|  | T2 | 0.71 (0.06, 8.31) | 0.63 (0.05, 8.04) |
|  | T3 | 0.81 (0.33, 2.04) | 0.83 (0.33, 2.08) |
| PFHxS |  |  |  |
|  | T1 | 1.38 (0.28, 6.87) | 1.02 (0.20, 5.18) |
|  | T2 | 0.38 (0.01, 10.50) | 0.35 (0.01, 10.08) |
|  | T3 | 1.10 (0.52, 2.32) | 1.25 (0.59, 2.67) |
| PFHpA |  |  |  |
|  | T1 | 0.96 (0.51, 1.82) | 0.95 (0.50, 1.81) |
|  | T2 | 1.27 (0.18, 8.54) | 1.57 (0.23, 10.57) |
|  | T3 | 1.30 (0.73, 2.31) | 1.34 (0.74, 2.45) |
| PFBS |  |  |  |
|  | T1 | 0.89 (0.31, 2.54) | 0.90 (0.31, 2.60) |
|  | T2 | 1.99 (0.31, 12.74) | 1.95 (0.30, 12.58) |
|  | T3 | 0.61 (0.26, 1.47) | 0.58 (0.24, 1.40) |
| PFDoA |  |  |  |
|  | T1 | 0.64 (0.16, 2.55) | 0.65 (0.16, 2.72) |
|  | T2 | 1.20 (0.17, 8.40) | 1.24 (0.17, 9.06) |
|  | T3 | 0.85 (0.29, 2.51) | 0.79 (0.26, 2.42) |

*Adjusted for maternal age (years), pre-pregnancy BMI (kg/m^2^), parity (0, ≥1, unknown), parental educational levels (≤12, >12 years), pregnancy complicating with chronic diseases (no, yes), infant sex (male, female) and gestational age at blood drawn (weeks).

Table S10. Associations between PFAS concentrations in early pregnancy and preterm birth with adjustment of education in finer categories (≤12, 12 -16, >16 years).

| PFAS (ng/ml) | Overall preterm birth | Spontaneous preterm birth | | Indicated preterm birth |
| --- | --- | --- | --- | --- |
|  | N_preterm_=136/N_term_2713 | | N_preterm_=97/N_term_=2713 | N_preterm_=39/N_term_=2713 |
|  | aOR* (95% CI) | | aOR* (95% CI) | aOR* (95% CI) |
| PFOA | 0.95 (0.62, 1.44) | | 0.77 (0.48, 1.25) | 1.62 (0.75, 3.52) |
| PFNA | 0.89 (0.63, 1.25) | | 0.88 (0.59, 1.31) | 0.92 (0.48, 1.75) |
| PFDA | 0.91 (0.68, 1.21) | | 0.85 (0.61, 1.12) | 1.08 (0.63, 1.86) |
| PFUA | 0.84 (0.62, 1.13) | | 0.83 (0.58, 1.17) | 0.88 (0.50, 1.54) |
| PFOS | 0.87 (0.64, 1.19) | | 0.80 (0.55, 1.16) | 1.09 (0.61, 1.93) |
| PFHxS | 1.16 (0.79, 1.70) | | 1.02 (0.65, 1.62) | 1.58 (0.81, 3.10) |
| PFHpA | 0.96 (0.77, 1.20) | | 0.92 (0.71, 1.19) | 1.06 (0.71, 1.58) |
| PFBS | 1.07 (0.83, 1.37) | | 0.93 (0.69, 1.26) | 1.44 (0.92, 2.24) |
| PFDoA | 0.72 (0.50, 1.03) | | 0.69 (0.46, 1.05) | 0.82 (0.43, 1.56) |

Note: PFAS concentrations were measured in maternal plasma and have been ln-transformed before entering into the model.

* Adjusted for maternal age (years), pre-pregnancy BMI (kg/m^2^), parity (0, ≥1), parental educational levels (≤12, 12 -16, >16, years), pregnancy complicating with chronic diseases (no, yes), infant sex (male, female) and gestational age at blood drawn (weeks).

Table S11. Associations between PFAS concentrations in early pregnancy and late preterm birth (34 - 36 weeks).

| PFAS (ng/ml) | Late preterm birth | | Spontaneous late preterm birth | | Indicated late preterm birth | |
| --- | --- | --- | --- | --- | --- | --- |
|  | N_preterm_=115/N_term_2713 | | N_preterm_=81/N_term_=2713 | | N_preterm_=34/N_term_=2713 | |
|  | aOR (95% CI) | aOR* (95% CI) | aOR (95% CI) | aOR* (95% CI) | aOR (95% CI) | aOR* (95% CI) |
| PFOA | 1.00 (0.64, 1.55) | 0.98 (0.63, 1.53) | 0.82 (0.48, 1.39) | 0.81 (0.48, 1.36) | 1.58 (0.71, 3.48) | 1.59 (0.70, 3.57) |
| PFNA | 0.79 (0.56, 1.13) | 0.79 (0.55, 1.13) | 0.76 (0.50, 1.16) | 0.76 (0.50, 1.16) | 0.87 (0.46, 1.64) | 0.89 (0.46, 1.72) |
| PFDA | 0.84 (0.62, 1.13) | 0.85 (0.63, 1.15) | 0.76 (0.53, 1.08) | 0.76 (0.53, 1.11) | 1.06 (0.61, 1.82) | 1.14 (0.65, 2.00) |
| PFUA | 0.77 (0.57, 1.06) | 0.79 (0.58, 1.08) | 0.75 (0.52, 1.08) | 0.74 (0.51, 1.08) | 0.85 (0.48, 1.48) | 0.94 (0.52, 1.68) |
| PFOS | 0.84 (0.60, 1.18) | 0.83 (0.59, 1.16) | 0.75 (0.50, 1.11) | 0.73 (0.49, 1.09) | 1.12 (0.62, 2.03) | 1.15 (0.63, 2.11) |
| PFHxS | 1.16 (0.77, 1.74) | 1.14 (0.75, 1.82) | 1.04 (0.65, 1.70) | 1.00 (0.61, 1.66) | 1.46 (0.75, 2.90) | 1.54 (0.76, 3.12) |
| PFHpA | 1.00 (0.79, 1.25) | 0.97 (0.77, 1.23) | 0.96 (0.73, 1.25) | 0.93 (0.70, 1.24) | 1.10 (0.73, 1.65) | 1.07 (0.70, 1.65) |
| PFBS | 1.12 (0.86, 1.45) | 1.10 (0.85, 1.44) | 0.97 (0.71, 1.33) | 0.96 (0.70, 1.32) | 1.53 (0.97, 2.43) | 1.51 (0.95, 2.40) |
| PFDoA | 0.73 (0.51, 1.06) | 0.72 (0.50, 1.05) | 0.75 (0.49, 1.16) | 0.71 (0.46, 1.10) | 0.69 (0.36, 1.32) | 0.75 (0.38, 1.47) |

Note: PFAS concentrations were measured in maternal plasma and have been ln-transformed before entering into the model.

* Adjusted for maternal age (years), pre-pregnancy BMI (kg/m^2^), parity (0, ≥1), parental educational levels (≤12, >12 years), pregnancy complicating with chronic diseases (no, yes), infant sex (male, female) and gestational age at blood drawn (weeks).
